# Remineralization Induced by Biomimetic Hydroxyapatite Toothpastes on Human Enamel

**DOI:** 10.3390/biomimetics8060450

**Published:** 2023-09-23

**Authors:** Alexandra-Diana Florea, Lucian Cristian Pop, Horea-Rares-Ciprian Benea, Gheorghe Tomoaia, Csaba-Pal Racz, Aurora Mocanu, Cristina-Teodora Dobrota, Reka Balint, Olga Soritau, Maria Tomoaia-Cotisel

**Affiliations:** 1 Research Center of Physical Chemistry, Faculty of Chemistry and Chemical Engineering, Babeş-Bolyai University, 11 Arany Janos Str., 400028 Cluj-Napoca, Romania; diana_florea03@yahoo.com (A.-D.F.); lucian.pop@ubbcluj.ro (L.C.P.); csaba.racz@ubbcluj.ro (C.-P.R.); aurora.mocanu@ubbcluj.ro (A.M.); cristina.dobrota@ubbcluj.ro (C.-T.D.); reka.balint@ubbcluj.ro (R.B.); 2 Department of Orthopedics and Traumatology, Iuliu Hatieganu University of Medicine and Pharmacy, 47 Gen. Traian Mosoiu Str., 400132 Cluj-Napoca, Romania; beneahorea@yahoo.com (H.-R.-C.B.); tomoaia2000@yahoo.com (G.T.); 3 Academy of Romanian Scientists, 3 Ilfov Str., 050044 Bucharest, Romania; 4 Department of Molecular Biology and Biotechnology, Faculty of Biology and Geology, Babeş-Bolyai University, 44 Republicii Str., 400015 Cluj-Napoca, Romania; 5 Oncology Institute of Cluj-Napoca, 34-36 Republicii Str., 400015 Cluj-Napoca, Romania

**Keywords:** atomic force microscope (AFM), crystallinity, demineralization, enamel, hydroxyapatite, surface roughness, X-ray diffraction (XRD)

## Abstract

This work aimed to compare the effect of four new toothpastes (P1–P4) based on pure and biomimetic substituted nano-hydroxyapatites (HAPs) on remineralization of human enamel. Artificially demineralized enamel slices were daily treated for ten days with different toothpastes according to the experimental design. Tooth enamel surfaces were investigated using atomic force microscope (AFM) images and surface roughness (Ra) determined before and after treatment. The surface roughness of enamel slices was statistically analyzed by one-way ANOVA and Bonferroni’s multiple comparison test. X-ray diffraction (XRD) and Fourier transform infrared (FTIR) data revealed the HAP structure with crystal sizes between 28 and 33 nm and crystallinity between 29 and 37%. The average size of HAP particles was found to be between 30 and 40 nm. The Ra values indicated that P3 (HAP-Mg-Zn-Sr-Si) toothpaste was the most effective after 10 days of treatment, leading to the lowest mean roughness. The P3 and P2 (HAP) toothpastes were found to be effective in promoting remineralization. Specifically, their effectiveness can be ranked as follows: P3 = P2 > P4 (HAP-Mg-Zn-Si) > P1 (HAP-Zn), considering both the chemical composition and the size of their constitutive nanoparticles. The proposed toothpastes might be used successfully to treat early tooth decay.

## 1. Introduction

Human caries is a disease for which a cure has been sought since the earliest days of mankind [1,2,3]. Dental caries, referred to as tooth decay cavities or simply caries, is caused by bacteria that demineralize and destroy the tooth’s hard tissues, including enamel, dentin, and cementum. If left untreated, the decay can progress into the tooth, causing pain and infection, which can eventually lead to tooth loss [4,5,6,7].

Tooth enamel is the outermost layer of teeth and protects them from deterioration. However, enamel can erode due to factors such as acidic foods or poor oral hygiene [8]. Toothpastes are essential for maintaining oral hygiene and preventing dental problems [9]. Developing new toothpastes with advanced formulations can enhance their effectiveness in preventing cavities. Biomimetic toothpastes replicate the natural processes and structures of teeth and can help improve oral health by promoting the remineralization of tooth enamel, strengthening the teeth, and reducing the risk of dental caries. These formulations can aid in the treatment of early-stage tooth decay and prevent further damage [10,11,12,13,14,15].

Synthetic stoichiometric hydroxyapatite (HAP) is one of the materials being researched in this area [16,17]. Its composition differs from that of biological hydroxyapatite in that HAP contains ionic substitutions within its lattice, such as Mg^2+^, Na^+^, and CO_3_^2−^, just to name a few. Since the 1950s, hydroxyapatite, the mineral that is the principal component of human bone and teeth, together with some organic components and water, has been widely researched in regenerative science as a material for numerous biomedical applications [18,19]. It is considered biocompatible because its chemical composition is similar to the mineral components of hard tissue and because it is well tolerated by living tissue without causing adverse reactions [20,21].

Different HAP-containing toothpastes have been developed to (i) minimize tooth sensitivity, which causes discomfort when eating food at temperatures quite different from the temperature of the tooth, by forming a protective layer over exposed dentin, thus reducing the transmission of external stimuli to the nerves in the teeth [22]; (ii) control dental plaque, a biofilm that forms on the teeth and contributes to the development of tooth decay by inhibiting the growth and attachment of harmful bacteria, reducing the risk of oral diseases [23]; and (iii) remove surface stains from the tooth by being a mild abrasive, thus being employed as a tooth whitening ingredient [24].

Hydroxyapatite is used as such in toothpastes [25,26,27] and bone regeneration [28,29], or as substituted in its lattice with various physiological elements to enhance its bioactivity, like Zn [30], Zn-carbonate [31], Sr [32,33], Zn and Sr [34], Cu and Zn [35], Zn and fluoride [36], fluorine [37], Ag and fluoride [38], Mg and Sr [39], Mg [40], Mg and Zn [41], Mg and Si [42], Ag [43], and Si [44]. These components play various roles; for some, the particular role they play in the substituted HAP is known; for others, it is only assumed. Even if the substitution is minor, it might alter the space group, morphology, and stability properties of the substituted HAP.

Zinc-substituted hydroxyapatite generally exhibits good biocompatibility because zinc ions can interact with surrounding tissues, promoting biomineralization and bone regeneration processes. It also has bactericidal and fungicidal properties and has been described to promote cell proliferation, differentiation, and mineralization [30]. In magnesium-substituted hydroxyapatite, Mg^2+^ replaces a portion of Ca^2+^ in the HAP lattice, which alters both the crystal structure and properties of HAP, which in turn influences the material’s chemical stability, namely its dissolution behavior and biocompatibility. Furthermore, these ions help suppress acid-producing bacteria, lowering their potential to induce tooth decay. They may also ease inflammation while reducing tooth sensitivity by blocking exposed dentinal tubules [45]. As in the case of Mg-HAP, strontium can be a substitute for a part of the calcium ions in the HAP structure. Strontium has been shown to possess some desensitizing properties by blocking or reducing the transmission of nerve impulses in the dentin and remineralization properties, thus strengthening the enamel and helping to repair the early stages of tooth decay [46]. When silicon is substituted into the hydroxyapatite structure, the resulting material exhibits enhanced remineralization properties and increased resistance to acid attacks without causing any harm or irritation to the teeth or gums [47].

An ideal toothpaste should be nontoxic, non-irritating, and not overly abrasive, with the primary goal of preventing tooth decay and biofilm formation [48,49]. Starting from all of this, we designed and prepared four toothpastes: one containing nano-hydroxyapatite (HAP) and three containing substituted HAPs, namely Zn-HAP, Zn-Mg-Si-HAP, and Zn-Mg-Sr-Si-HAP. Thus, we used ingredients that are more biocompatible with oral tissues, reducing the risk of irritation or allergic reactions.

Typically, a toothpaste should contain several main components: binders used to keep the solid and liquid phases together, while preventing the toothpaste from drying out and conferring adequate viscosity; anticaries agents, which, as the name suggests, are powerful anticaries tools; antiplaque agents used to remove the plaque; abrasives with the role of mechanically removing stains from teeth; foaming agents (surfactants), with their ability to lower surface tension facilitating the contact between the teeth and the toothpaste contents and the dissolution of dental plaque; whitening agents capable of increasing the whiteness of teeth by abrasion; sweeteners to give a pleasant taste to the paste; and, of course, water as a solvent [50,51]. Also, depending on the specific toothpaste, it may contain desensitizing compounds, anti-halitosis agents, other pharmaceutical agents, preservatives, flavoring and coloring agents, etc.

In our previous work, monosubstituted HAP (HAP-Zn [52]) and multisubstituted HAP (i.e., HAP-Mg-Zn-Si [53,54] and HAP-Mg-Zn-Sr-Si [55,56]) samples were synthesized and characterized. The ions released from these multisubstituted HAPs (ms-HAPs) were examined in simulated body fluid and displayed a good relationship as a function of the amount of Mg, Zn, Sr, and Si incorporated into ms-HAPs and the ion release time. Among all the synthesized nanomaterials, HAP-Zn, HAP-Mg-Zn-Si, and HAP-Mg-Zn-Sr-Si seem to provide a good balance of properties for bone regeneration and osseointegration [53,55,56,57].

According to published research, there is a gap in the development of toothpastes containing ms-HAP, which could have an enhanced outcome in the remineralization of enamel. Therefore, in this study, we extend our previous work by developing new ms-HAPs to control their structure and crystallinity, as well as the shape and size of the nanoparticles, while also assessing their biomimetic mineralization of enamel.

## 2. Materials and Methods

### 2.1. Materials

Nitrates were purchased from Sigma-Aldrich: Ca(NO_3_)_2_·4H_2_O (calcium nitrate tetrahydrate, >99%), Mg(NO_3_)_2_·6H_2_O (magnesium nitrate hexahydrate, 99%), Zn(NO_3_)_2_·6H_2_O (zinc nitrate hexahydrate, >98%), and Sr(NO_3_)_2_ (strontium nitrate, 99.995%). Also, sorbitol (≥98%), polyethylene glycol (PEG 400), sodium dodecyl sulfate (≥99.0%), SiO_2_ (silicon dioxide, nanopowder, 10–20 nm particle size, 99.5%), H_3_PO_4_ (orthophosphoric acid, 85 wt% in H_2_O), and xanthan gum were bought from Sigma-Aldrich. (NH_4_)_2_HPO_4_ (diammonium hydrogen phosphate, >99%) and ammonia solution (NH_4_OH, 25%) were purchased from Chempur, and tetraethyl orthosilicate (TEOS 98%) was purchased from Alfa Aesar. All substances were used as received without additional purification.

### 2.2. Synthesis of Four HAPs Used in Toothpastes

Pure HAP and the substituted HAPs were prepared using a wet chemical route, starting from a solution containing the necessary cations and another with specific anions, according to the composition to be attained [56,58]. The first solution was prepared from the corresponding nitrates dissolved in ultrapure water to achieve a total cation concentration of 0.25 M using Ca(NO_3_)_2_∙4H_2_O for pure HAP (P2 paste) and for all the substituted HAPs and Zn(NO_3_)_2∙_6H_2_O (for HAP-5% Zn, P1, and also for P3 and P4), together with Mg(NO_3_)_2∙_6H_2_O (for P4) and with Sr(NO_3_)_2_ (for P3). The 0.15 M anion aqueous solution consisted of PO_4_^3−^ and (for P3 and P4) also SiO_4_^4-^, obtained from (NH_4_)_2_HPO_4_ and TEOS, respectively, in the calculated ratio. The adequate working pH was established at 11.5 with the aid of an ammonia solution. The two solutions (equal volumes, to maintain the mole ratio (Ca+Mg+Zn+Sr)/(P+Si) at the theoretical value for HAPs, 5/3) were quickly mixed at an ambient temperature of 22 °C. The acquired suspension underwent two stages of maturation, the first one at approximately 22 °C/24 h and the second one at 70 °C/24 h under discontinuous mixing. The obtained precipitate was filtered (using a grade 389 Munktell filter, 8–12 m pore size, 150 mm diameter, 84 g/m^2^). Then, at room temperature, it was rinsed repetitively with ultrapure water until it was nitrate-free, followed by lyophilization, and ground to a fine powder using a ball mill.

### 2.3. Preparation of Toothpastes

The development of new experimental toothpastes is a complex procedure that necessitates several technological steps. These are connected to the manufacture of an aqueous suspension, which requires the exact mixing of several components.

Preparation for 100 g of toothpaste: Step I in this case entails combining a specific amount of silica dioxide (9.00 g) with a precise amount of distilled water (27.67 g). After resting for 25 min, the suspension is vigorously homogenized in a sealed container before resting for another half hour. Step II involves preparing an aqueous suspension by dispersing hydroxyapatite (3.70 g) in an exact amount of distilled water (24.33 g). This is followed by 50 min of mixing with a magnetic stirrer (200 rpm). The hydrated silica dioxide is then added while constantly swirling until thoroughly homogenized. Step III involves combining sorbitol (7.35 g) with an exact amount of distilled water (20.0 g), to which PEG 400 (7.35 g) and xanthan gum (0.40 g) are added. The mixture is homogenized until it forms a fine, white paste. Step IV: The paste from step III is vigorously mixed with the mixture from step II. The mixture is stirred for about 8–10 min, and then sodium dodecyl sulfate (0.20 g) is added. The prepared toothpastes were used to remineralize the artificially demineralized dental enamel.

### 2.4. Study Protocol for Obtaining Enamel Slices

The study protocol and all the procedures were approved by the Ethics Committee of “Iuliu Hatieganu” University of Medicine and Pharmacy (UMP), Cluj-Napoca (Approval No. 85/19 July 2017). Eighteen healthy adult third molars removed for orthodontic purposes were used in this investigation. The lack of cracks, the lack of hypoplastic or carious lesions, and the lack of restorations on the molar surfaces were the selection criteria. For 5 min, the extracted molars were ultrasonically cleaned of soft tissue debris and stored in deionized water. The qualitative evaluation of the enamel surface was performed through a clinical examination using a stereomicroscope (Carl Zeiss Stereo 475002, Gottingen, Germany). Third molars were placed within auto-polymerizing acrylate prisms (Duracryl Plus, Spofadental Inc., Jin, Czech Republic) to enhance sample handling, while the coronal part was left exposed.

Using a microtome (Microtome IsoMet^®^), longitudinal enamel slices with dimensions of 8 mm × 6 mm and a thickness of 1.5 mm were sectioned from the buccal and lingual surface of every third molar specimen. A total of 36 enamel samples were collected and divided into two groups: one control group (Ctrl), with *n* = 6 slices of natural enamel that did not receive any treatment, and another group of 30 enamel slices that were artificially demineralized for 60 s using orthophosphoric acid 37.5% (Gel Etchant, Kerr Dental, Orange, CA, USA). They were washed for half a minute with ultrapure water to neutralize and eliminate compounds present on the tooth enamel surface and divided into two groups: the negative control group (the NC group with *n* = 6 demineralized samples, which were deposited into deionized water), and the group of 24 demineralized slices which were divided equally into the four test groups, with each test group having *n* = 6 demineralized enamel slices. These demineralized enamel slices were treated with toothpastes; the P1 test group was treated with P1 toothpaste, the P2 test group was treated with P2 toothpaste, the P3 test group was treated with P3 toothpaste, and the P4 test group was treated with P4 toothpaste. The sample size was established using the same approach as in related studies on the subject [59,60,61,62].

### 2.5. Enamel Treatment with Toothpaste

For 10 days, a certain toothpaste of those tested (P1–P4) was applied to a particular test group (P1–P4) of enamel slices. The samples were brushed in circular motions with a brush applicator (3M^TM^ Applicator Handles and Disposable Applicator Brush Tips, Corona, CA, USA). The treatment consisted of brushing the demineralized enamel slices for 3 min twice a day (morning and evening), followed by gentle cleaning with distilled water and storage in deionized water. The collected samples were stored in sterile PET containers with screw-on lids, in deionized water. Before the AFM measurements were performed, the samples were cleansed with an ultrasonic cleaner for 5 min and dried. Then, the samples were investigated.

### 2.6. Methods

X-ray diffraction (XRD) investigations were conducted using a DRON-3 diffractometer in Bragg–Brentano geometry with an X-ray tube containing cobalt Kα radiation, wavelength 1.79026 Å, 25 kV/20 mA. The XRD powder patterns were collected using a 2° angle scale (10–80°) with a step size of 0.02° and a normalized count time of 1 s/step to 2 s/step.

FT-IR spectra were measured on KBr pellets containing the HAP powders (0.5 wt%) using a JASCO 6100 FT-IR spectrometer in the 4000–400 cm^−1^ range of wavenumbers (resolution 4 cm^−1^).

To analyze the nanostructure of the HAP samples, a Hitachi SU-8230 field emission scanning electron microscope (FE-SEM or SEM) operated at 30 kV was employed. For HAP elemental analysis, an FE-SEM equipped with an Oxford energy-dispersive X-ray spectrometer (EDS) was employed (energy-dispersive X-ray spectroscopy (EDX) spectra). Carbon coatings ranging in thickness from 10 to 20 nm were applied to Cu SEM grids. Powdered HAP particles were deposited in thin layers on SEM grids to make SEM samples. A Hitachi HD-2700 scanning transmission electron microscope (STEM) operating at 200 kV and 30 kV was also used.

An OPTIMA 5300DV inductively coupled plasma optical emission spectrometer (ICP-OES) (Perkin-Elmer, Waltham, MA, USA) was used for the elemental analysis.

Images were obtained using an AFM JEOL 4210 instrument in tapping mode with traditional cantilevers with silicon nitride tips (resonant frequency 200–300 kHz, spring constant 17.5 N/m) [63,64,65]. After being dispersed, the particles were adsorbed onto an optically polished glass support. The dispersion of HAPs in water used for AFM imaging was homogenized using a high-intensity ultrasonic device (Sonics Vibra-Cell).

Surface analysis was performed after 10 days of enamel treatment with toothpaste to obtain the following data: Ra (mean arithmetic roughness). The results were analyzed using GraphPad Prism 5 software, 5.0 applying one-way ANOVA analysis followed by Bonferroni’s multiple comparison test.

## 3. Results

Table 1 lists the four hydroxyapatites and substituted hydroxyapatites, as well as their theoretical formulas.

**Figure 1:**
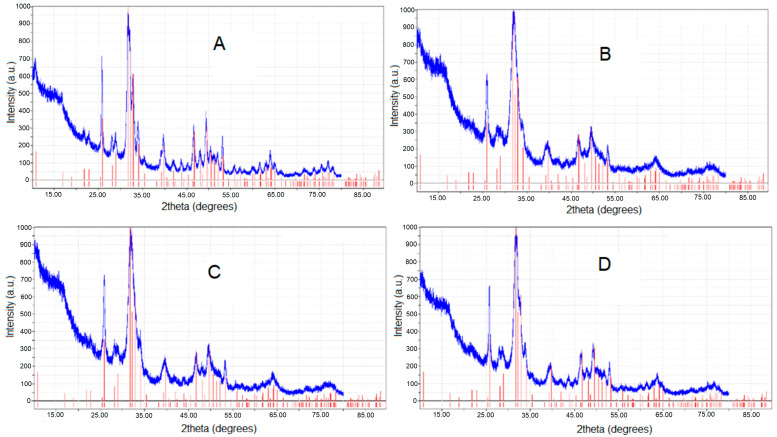
XRD for four HAPs: (**A**) for hydroxyapatite, HAP, Ca_10_(PO_4_)_6_(OH)_2_; (**B**) for HAP-Zn, Ca_9.22_Zn_0.78_(PO_4_)_6_(OH)_2_; (**C**) for HAP-Mg-Zn-Si, Ca_8.80_Mg_1.00_Zn_0.20_(PO_4_)_5.00_(SiO_4_)_1.00_ (OH)_1.00_; and (**D**) for HAP-Mg-Zn-Sr-Si, Ca_8.19_Mg_0.10_Zn_0.50_.Sr_1.21_(PO_4_)_5.25_(SiO_4_)_0.75_(OH)_1.25_, compared with PDF:74-0566.

The XRD patterns for the four HAPs used are given in Figure 1, along with the pattern for pure HAP from PDF:74-0566 (red vertical lines). The calculated lattice parameters (*a = b* and *c* values), crystallite sizes, and crystallinity degree are included in Table 2, compared with average NP (nm) diameters estimated from AFM images.

The calculated lattice parameters (*a* and *c* values) revealed only slight changes with the compositional modification within the HAP structure. The small composition differences lead to a slight distortion of the HAP lattice and, thus, a small drop in its crystallinity (Table 2). It was also discovered that the lattice constants, *a* and *c*, diminished slightly with Zn substitution in the HAP structure.

**Figure 2:**
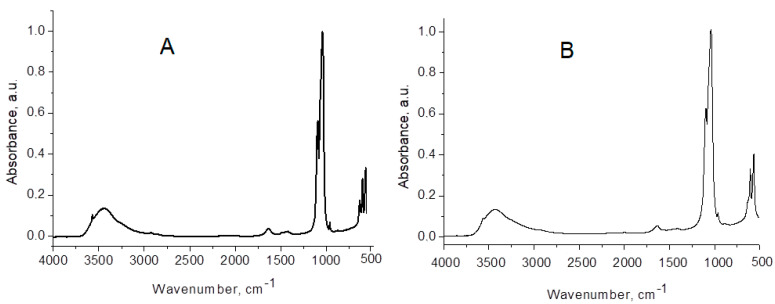
FTIR spectra of lyophilized HAP (**A**), used in P2 toothpaste, and of HAP−Mg−Zn−Sr−Si (**B**), used in toothpaste P3. Absorbance is normalized to “1”.

Figure 2 presents the experimental FTIR spectra of lyophilized HAP (A) and HAP-Mg-Zn-Sr-Si (B), both used in our toothpastes, The wavenumbers (cm^−1^) of absorption peaks and the assignments of the corresponding vibrations are given in Table 3.

**Figure 3:**
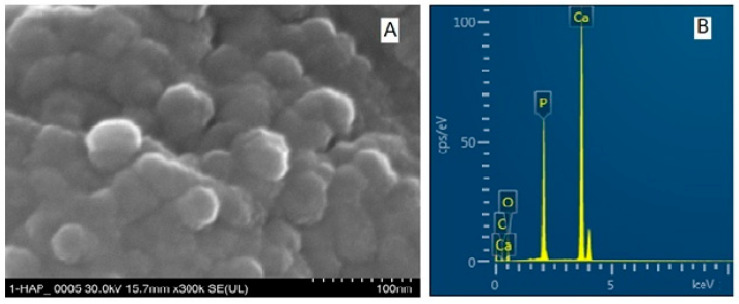
FE-SEM image (**A**) for HAP; the bar scale is 100 nm. EDX spectrum (**B**) jointly showing all elements in FE-SEM image (**A**).

For SEM-EDX measurement, the powder HAP samples were deposited in slim sheets on SEM grids. An FE-SEM image (Figure 3A) shows individual particles at high magnification. The average diameter of HAP particles was found to be 40.0 ± 7.5 nm.

**Figure 4:**
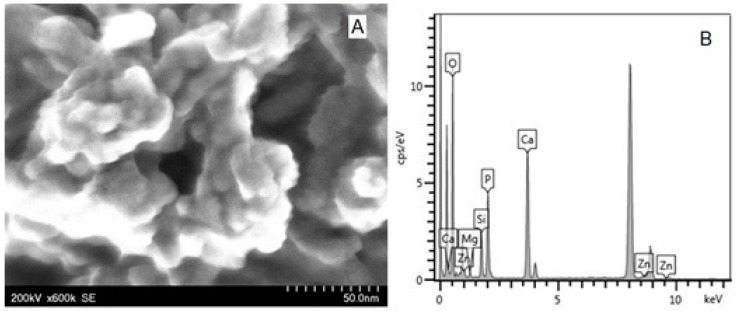
STEM image of HAP-Mg-Zn-Si (**A**) and EDX spectrum (**B**) showing all the elements existent in the sample.

Figure 4A shows the STEM image of the self-assembled trisubstituted HAP nanoparticles, and Figure 4B shows that, in addition to the elements present in the pure HAP, the elements with which the HAP has been doped also appear.

**Figure 5:**
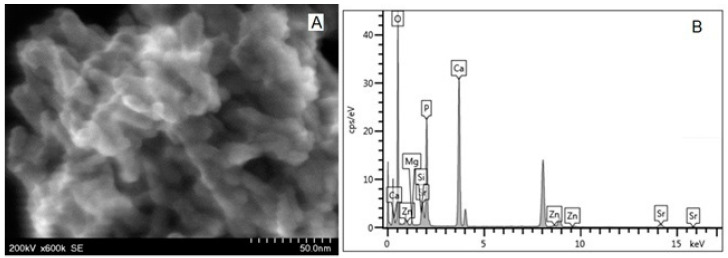
STEM image (**A**) and EDX spectrum (**B**) for HAP-Mg-Zn-Sr-Si.

Figure 5A shows the STEM image of the associated tetrasubstituted HAP nanoparticles used in the P3 paste, and Figure 5B shows the EDX spectrum where all the constituents of the doped HAP can be seen.

**Figure 6:**
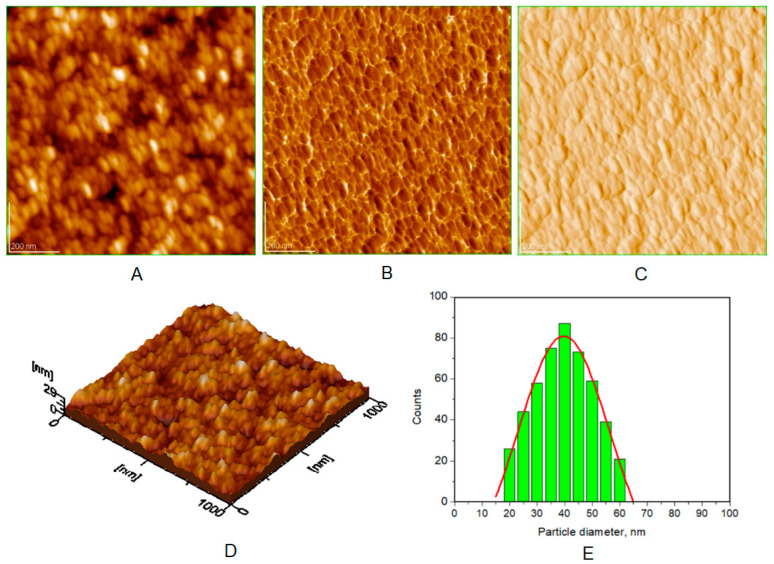
AFM images of HAP-5% Zn: (**A**) topography image, (**B**) phase image, (**C**) amplitude image, (**D**) 3D image, and (**E**) histogram for image (**A**). Scanned area 1 μm × 1 μm. Particle diameter is determined from histograms (at least 3) as 40 ± 5 nm.

**Figure 7:**
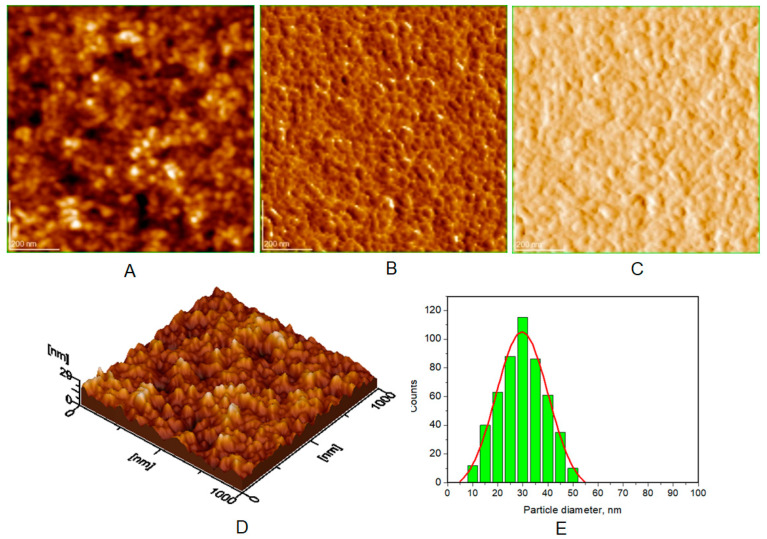
AFM images of HAP: (**A**) topography image, (**B**) phase image, (**C**) amplitude image, (**D**) 3D image, and (**E**) histogram for image (**A**). Scanned area 1 μm × 1 μm. Particle diameter is determined from histograms (**E**) (at least 3) as 30 ± 3 nm.

**Figure 8:**
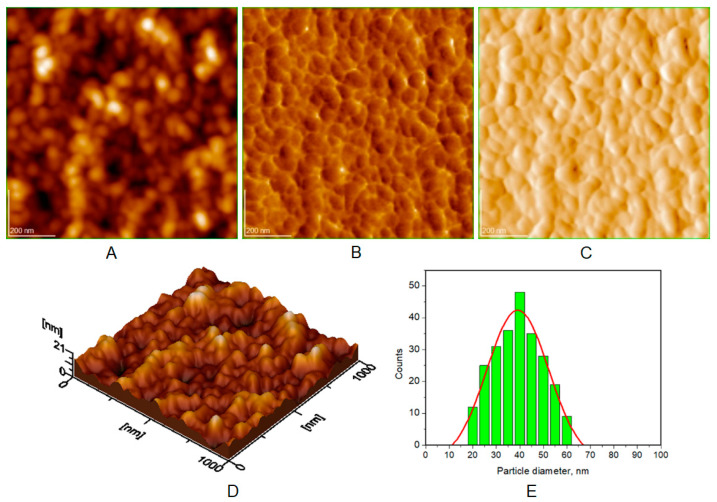
AFM images of HAP-Mg-Zn-Sr-Si (HAP-0.23%Mg-3.09%Zn-10%Sr-2%Si): (**A**) topography image, (**B**) phase image, (**C**) amplitude image, (**D**) 3D image, and (**E**) histogram for image (**A**). Scanned area 1 μm × 1 μm. Particle diameter is determined from histograms (**E**) (at least 3) as 37 ± 4 nm.

**Figure 9:**
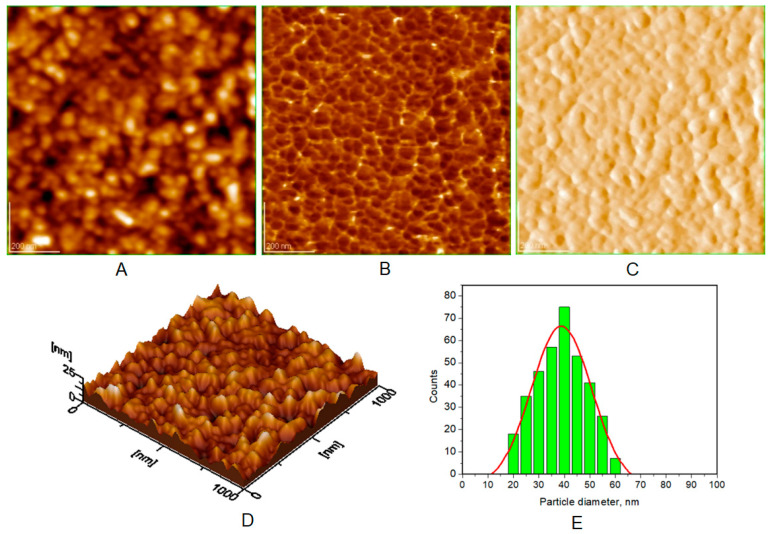
AFM images of HAP-Mg-Zn-Si (HAP-2.50%Mg-1.34%Zn-2.90%Si): (**A**) topography image, (**B**) phase image, (**C**) amplitude image, (**D**) 3D image, and (**E**) histogram for image (**A**). Scanned area 1 μm × 1 μm. Particle diameter is determined from histograms (**E**) (at least 3) as 38 ± 5 nm.

**Figure 10:**
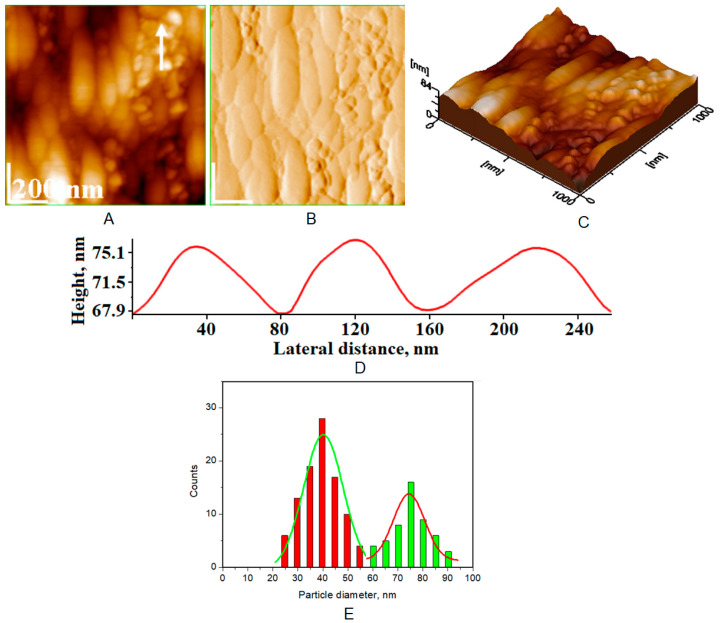
AFM images of two half-slices: one half-slice was untreated (natural enamel for control) and is marked with a white arrow, and the other half was demineralized by treatment with phosphoric acid: (**A**) topography image; (**B**) amplitude image; (**C**) 3D image; (**D**) cross-section profile for white arrow in image (**A**), representing natural enamel zone; and (**E**) histogram for image (**A**). Average diameter of NPs was 42 ± 5 nm on natural enamel and 73 ± 6 nm on demineralized enamel. Scanned area 1 μm × 1 μm.

**Figure 11:**
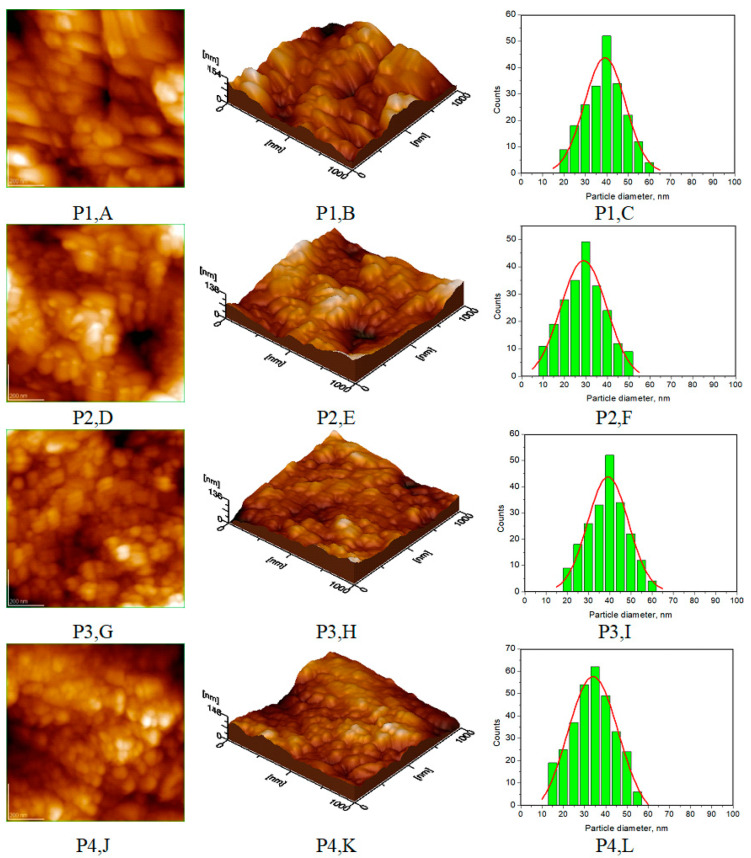
AFM images: 2D topography (**A**,**D**,**G**,**J**), 3D topography (**B**,**E**,**H**,**K**), and histograms for images (**C**,**F**,**I**,**L**) for demineralized enamel treated for 10 days with four toothpastes: P1 (**A**–**C**), P2 (**D**–**F**), P3 (**G**–**I**), and P4 (**J**–**L**), for scanned area of 1 µm × 1 µm.

Atomic force microscopy (AFM) images were collected for all the prepared HAP samples (Figure 6, Figure 7, Figure 8 and Figure 9), as well as all teeth, both unmineralized and demineralized with phosphoric acid (Figure 10), and those treated with the newly developed toothpaste (Figure 11). On optically polished glass plates, the particles were adsorbed from their aqueous dispersion; then, in all cases, the area scanned was 1 μm × 1 μm.

It can be noted that the particles in HAP samples are spherical or oval, and their sizes range from 30 nm for unsubstituted HAP (Figure 7) to 40 nm for HAP-5% Zn (Figure 6), values close to those of natural enamel (42 nm, Figure 10E). These values are fairly similar to those obtained by XRD (Table 2). In the case of artificially demineralized teeth (Figure 10), there is not only an increase in roughness (Ra = 9.92 nm) but also an increase in the average particle size (73 nm).

Figure 11 shows AFM images of a remineralized enamel surface treated with P1 to P4 toothpastes for 10 days, showing 2D topography (A,D,G,J), 3D topography (B,E,H,K), and histograms (C,F,I,L) for the 2D images for artificially demineralized enamel treated for 10 days with the four toothpastes P1 (A–C), P2 (D–F), P3 (G–I), and P4 (J–L), for scanned area of 1 µm × 1 µm.

These images show the surface morphology of the enamel surface, confirming the globular nature of HAP nanoparticles that are uniformly dispersed across the enamel surface with an average diameter of about 40 nm for enamel treated with P1 (Figure 11C), with an average diameter of around 30 nm for P2 (Figure 11F), with an average diameter of about 37 nm for P3 (Figure 11I), and with an average diameter of about 35 nm for P4 (Figure 11L).

**Figure 12:**
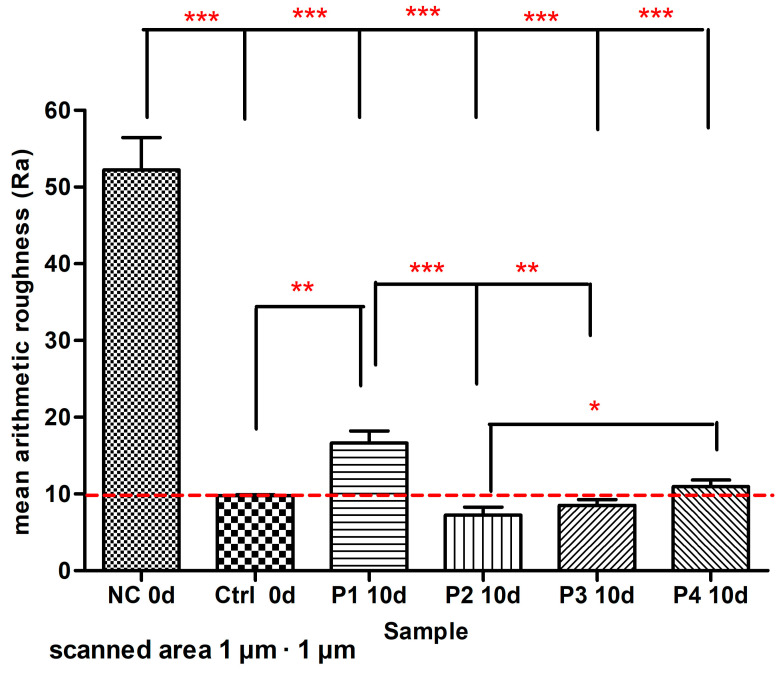
Ra values obtained in scanned 1 µm × 1 µm area for demineralized enamel surface (NC at 0 days), natural enamel (Ctrl) at 0 days and artificially demineralized surfaces treated with P1–P4 toothpastes for 10 days (10 d treatment). The degrees of statistical significance are marked by asterisks as follows: * 0.01 < *p* < 0.05; ** 0.001 < *p* < 0.01; *** *p* < 0.001.

In the surface roughness investigation employing one-way ANOVA and Bonferroni’s multiple comparison as a post-test of enamel samples, the scanned (1 µm × 1 µm) area revealed quite large variations between the artificially demineralized enamel (NC) and the natural enamel control. After 10 days of treatment with the four toothpastes, P1–P4, the lowest arithmetic mean roughness (Ra) was observed for P2 toothpaste containing nanostructured HAP, with Ra values that were not significantly different from control values in the statistical analysis, indicating a relative remineralization efficiency when compared to the corresponding Ra value of intact enamel. It was observed that Ra values for using P1–P4 toothpastes decline in the following order: P1 > P4 > Ctrl > P3 > P2 (Figure 12).

## 4. Discussion

Dental caries begins at the enamel level with the demineralization of the hard tissue caused by net mineral loss from the hydroxyapatite (HAP) lattice [66]. The disintegration of hydroxyapatite crystals in an acidic oral environment results in the loss of calcium and phosphate from the tooth enamel structure [25]. Biomimetic mineralization, which involves the attachment of a mineral layer to the surface of the teeth, is an excellent method for restoring the enamel structure [67].

This work provides a thorough examination of four novel nanomaterials utilized in the manufacture of toothpastes (Table 1). The toothpastes were prepared utilizing various substituted hydroxyapatites containing magnesium (Mg), zinc (Zn), strontium (Sr), and silicon (Si) as substitution elements. According to the literature, zinc (Zn^2+^), magnesium (Mg^2+^), and strontium (Sr^2+^) ions can be placed in the calcium position; SiO_4_^4−^ ions can be placed in the PO_4_^3−^ and hydroxyl position of hydroxyapatite; and carbonate (CO_3_^2−^) ions can be placed in the hydroxyl and phosphate positions [56], with a change in their mechanical properties accordingly [66].

The rationale for using these substitution elements in our toothpastes was that the hardness of enamel is highly correlated with the zinc content, the presence of which marks the beginning of biomineralization. Mg^2+^ ions can regulate HAP crystallization, hindering the growth of HAP crystals and thus forming new nano-HAP crystals [67]. Strontium demonstrates desensitizing and remineralization attributes, consequently bolstering enamel integrity [46]. Conversely, silicon manifests heightened remineralization characteristics, conferring resilience against acid-induced degradation [47].

The interactions between nano-HAP particles and the enamel surface that result in remineralization are not fully understood. One potential mechanism is that nano-HAP particles induce remineralization by acting as a nucleus that attracts calcium and phosphate from saliva [68].

X-ray diffraction (XRD) was used in our study to confirm the presence of a distinct HAP phase in both pure and substituted hydroxyapatites (Figure 1). Due to compositional modifications, lattice parameters *a* and *c* showed minor changes, with Zn substitution resulting in a little decrease in lattice constants (Table 2). The Ca (II) site in the HAP lattice delivered energetically favorable sites for Zn substitution. However, the ms-HAP lattice, both for three and four substitutions, exhibited a small increase in *a* and *c* values, indicating that there is a compensation effect of substitution of these elements within the HAP lattice leading to a stable ms-HAP lattice.

Table 3 provides an interpretation of the results acquired from the FTIR study. The FT-IR spectra of all samples (e.g., Figure 2) are very comparable, displaying all absorption peaks for the PO_4_^3−^, OH^-^, and CO_3_^2−^ groups in hydroxyapatites. FTIR spectra lack bands assigned to vibrations of non-apatitic HPO_4_^2−^ ions as well as peaks suggestive of nonstoichiometric apatites [56,69,70,71].

At high magnification, scanning electron microscopy (FE-SEM) images revealed individual hydroxyapatite particles (Figure 3A). According to the indicated composition, energy-dispersive X-ray spectroscopy (EDX) revealed the presence of calcium (Ca), phosphorous (P), and oxygen (O) elements for stoichiometric HAP (Figure 3B) and all substituting elements within ms-HAPs (STEM images and EDX spectra in Figure 4 and Figure 5). The average diameter of HAP particles was found from FE-SEM images (as in Figure 3A) to be 40.0 ± 7.5 nm. From the EDX spectra (as in the example given in Figure 3B), the concentrations of the elements are found to be in agreement with the theoretical composition of these elements determined by ICP-AOS. All the HAP components (Ca, P, and O) are visible in Figure 3B. Similar to this, the presence of all doping elements, specifically Mg, Zn, and Si, is confirmed from STEM images for multisubstituted HAPs employed in our toothpastes (Figure 4A and Figure 5A) and their EDX spectra (Figure 4B and Figure 5B).

AFM images were used to visualize the morphology and NP size characteristics for the pure HAPs used in the preparation of the four toothpastes (Figure 6, Figure 7, Figure 8 and Figure 9). The morphology of pure (natural) enamel and of artificially demineralized enamel is shown in Figure 10A–D, jointly with the histogram of their constitutive NPs (Figure 10E). Also, the morphological effects of treatment with the four toothpastes on the artificially demineralized enamel surface are given in Figure 11. Natural enamel contains 20–40 nm HAP nanoparticles, and it has been proposed that using 20 nm HAP nanoparticles is efficient in repairing damaged enamel [25]. In this study, HAP nanoparticles from demineralized enamel had a larger average size (73 nm) than those from naturally occurring enamel (42 nm), as seen in Figure 10E. This finding shows that biological HAP nanoparticles on the healthy enamel surface are smaller than those found inside the enamel due to surface erosion over time. Similar observations were previously shown [54,69,72].

Surface roughness (Figure 12) and particle size differences have been identified in AFM studies of natural enamel and demineralized enamel (Figure 10) and toothpaste-treated enamel (Figure 11). AFM images indicated round or oval nanoparticles with diameters ranging from 30 to 40 nm. Studies on surface roughness (Ra values) found that P2 toothpaste (containing HAP, Ca_10_(PO_4_)_6_(OH)_2_, with NPs of about 30 nm) had the highest remineralization efficacy and the lowest Ra corresponding values, comparable with P3 toothpaste (ms-HAP: Ca_8_._19_Mg_0_._10_Zn_0_._5_Sr_1_._21_(PO_4_)_5_._25_(SiO_4_)_0_._75_(OH)_1_._25_ with NPs of around 37 nm) indicating that both toothpastes have a close resemblance to natural enamel properties. These dimensions are similar to those found in natural enamel, meaning that the degree of remineralization achieved with these toothpastes would be comparable. Furthermore, these findings might be linked to the formation of an evenly distributed coating layer of synthetic HAP nanoparticles on the enamel surface, limiting the depths and types of lesions induced by the demineralizing process. These results are somewhat in agreement with existing evidence as described in previous studies [31,47,48,57,70,72,73]. Although the remineralization process is not completely understood, our findings suggest that the biomimetic hydroxyapatite nanoparticles adsorbed on the enamel surface can function as reservoirs for calcium and phosphate ions as well as for substituting ions in the HAP lattice. The released ions can contribute to biomimetic remineralization and regeneration of the enamel surface [56,57].

Statistical analysis of the roughness described by the mean Ra values from AFM imaging indicated (Figure 12) significant variations in roughness between NC and control with a *p* value of 0.0001 and between NC and all other surfaces treated with P1–P4 toothpastes with a *p* value of 0.0001. It should be noted that the P2 test group exhibited the lowest values when compared to all the P1, P3, and P4 groups, each of which was treated with the corresponding toothpaste, and even lower values than the control when compared to untreated intact enamel. This is explained by the use of smaller HAP nanoparticles (30 nm) to fill the smallest erosion cavity depths more efficiently. P1 toothpaste had the lowest remineralization efficiency with increasing Ra values, which was statistically significantly different from the control (*p* value 0.05), P2 (*p* value 0.01), and P3 (*p* value 0.05). The P3 and P4 remineralization efficiencies were about the same as the control value. As a result, P2 and P3 toothpastes shown a strong ability to repair artificially demineralized human enamel. The remineralization process has significant clinical value in the prevention and treatment of early stages of dental caries disease, and it is thus regarded as an important treatment technology in minimally invasive dentistry [67]. Nanosized HAP can facilitate HAP penetration into enamel pores (or microcracks); nanosized HAP fills the small cavities [73] and allows the addition of calcium and phosphate back into the enamel structure from supersaturated oral fluids and consequently may lead to its remineralization [25].

Changes in the concentration of substitution elements within the HAP lattice, as a future research direction, may allow for even more exact control over the enamel remineralization process. This potential advancement could lead to toothpastes tailored to various levels of enamel damage and sensitivity. Another approach could be to develop hydroxyapatite nanoparticles that accurately transport active compounds, such as antimicrobial or anti-inflammatory molecules [29]. These nanoparticles might be engineered to gradually release beneficial bio-compounds over time. This controlled release mechanism may give long-term protection against tooth decay, enamel demineralization, or gingivitis. As these new toothpaste formulations evolve, thorough biocompatibility and safety evaluations will become increasingly vital. Researchers will need to conduct thorough studies to explore the long-term consequences of nanoparticle exposure on oral tissues, overall systemic health, and potential interactions with existing dental therapies or medications. Finally, the future of hydroxyapatite-based toothpaste formulations holds enormous promise for personalized dental care, enhanced enamel remineralization, and the incorporation of cutting-edge technologies. Continued research and collaboration among dentists, materials scientists, and regulatory agencies will be critical in influencing these technical breakthroughs. We will be able to build a biomimetic remineralization technique by modeling the biomineralization process as our understanding of the biomineralization of dental hard tissues advances [66].

While these results provide valuable insights on enamel remineralization using nanomaterials, some limitations might be accepted to ensure an adequate comprehension of the implications of this research. This study is based on controlled laboratory conditions that may not fully reproduce the complex and dynamic oral environment. pH variations and bacterial activity can have a major impact on the efficiency of toothpaste formulations. This research work focuses on the short-term effects of toothpaste treatments, examining changes in enamel characteristics over a short period of time. In a future investigation, the strength of enamel remineralization will be considered jointly with the development of toothpaste containing supplementary antimicrobial components.

## 5. Conclusions

In conclusion, four biomimetic toothpastes (P1–P4) with a low concentration (3.7%) of pure hydroxyapatite or substituted hydroxyapatite and low crystallinity were developed with the potential to promote oral health and prevent tooth decay through enamel remineralization. The AFM study revealed that P3 toothpaste with tetrasubstituted hydroxyapatite (HAP-Mg-Zn-Sr-Si) performed the best in terms of human enamel remineralization when compared to the other toothpastes P1 (HAP-Zn) and P4 (HAP-Mg-Zn_Si), with approximately the same remineralization efficiency as P2 (HAP) toothpaste after 10 days of toothpaste treatment. The smallest nanosized particles (about 30 nm in average size of HAP) showed great potential in the remineralizing process by covering lesion regions of enamel. All results of treating human enamel for 10 days with each of the P1–P4 toothpastes lead us to assume that these toothpastes can be used successfully to treat early tooth decay and, more importantly, artificially demineralized enamel.

## Figures and Tables

**Table 1:** Four innovative nanomaterials used to prepare the four toothpastes.

Toothpaste Symbol	HAPs Type	Substitution Elements (wt%)	HAPs Chemical Formula
P1	HAP-Zn	Zn 5.00	Ca_9.22_Zn_0.78_(PO_4_)_6_(OH)_2_
P2	HAP	-	Ca_10_(PO_4_)_6_(OH)_2_
P3	HAP-Mg-Zn-Sr-Si	Mg 0.23 Zn 3.09 Sr 10.00 Si 2.00	Ca_8.19_Mg_0.10_Zn_0.5_Sr_1.21_(PO_4_)_5.25_(SiO_4_)_0.75_(OH)_1.25_
P4	HAP-Mg-Zn-Si	Mg 2.50 Zn 1.34 Si 2.90	Ca_8.80_Mg_1.00_Zn_0.20_(PO_4_)_5.00_(SiO_4_)_1.00_(OH)_1.00_

**Table 2:** XRD estimates of crystallite size, crystallinity degree, and lattice parameters for pure hydroxyapatite (HAP) and substituted hydroxyapatites: HAP-Zn, HAP-Mg-Zn-Si, and HAP-Mg-Zn-Sr-Si.

Hydroxyapatites	HAP-Zn	HAP	HAP-Mg-Zn-Sr-Si	HAP-Mg-Zn-Si
Toothpastes	P1	P2	P3	P4
Crystallites size (nm), from XRD data	30.3	33.1	28.2	30.6
Crystallinity (%), from XRD data	30.5	36.6	28.7	30.3
Lattice parameters:				
*a* = *b* (nm)	0.9421	0.9426	0.9466	0.9445
*c* (nm)	0.6862	0.6881	0.6904	0.6883
Average diameter of NPs (nm), from AFM approach *	40 ± 5	30 ± 3	37 ± 4	38 ± 5

* Average diameter of nanoparticles (NPs) self-assembled as a layer on a glass plate, estimated from AFM approach.

**Table 3:** Wavenumbers (cm^−1^) of IR absorption peaks and assignment of vibrations, from FTIR spectra of the samples.

HAP	HAP-Zn	HAP-Mg-Zn-Si	HAP-Mg-Zn-Sr-Si	Assignment of HAP Vibrations
3570	sh	sh	sh	stretching: structural O-H from HAP
3438	3437	3430	3430	O-H…O stretching: absorbed water with H-bonding
1635	1635	1632	1633	absorbed water bending mode ν_2_
-	-	1488	1489	CO_3_^2−^
1421	1407	1421	1420	CO_3_^2−^
1385	-	-	-	CO_3_^2−^
1094	1096	1096	1096	PO_4_ asymmetric stretching ν_3_
1043	1039	1039	1039	PO_4_ asymmetric stretching ν_3_
962	963	963	963	PO_4_ symmetric stretching ν_1_ (forbidden in IR)
875	-	874	-	CO_3_^2−^
634	sh	sh	sh	OH vibration
603	604	605	605	PO_4_ asymmetric bending ν_4_
567	566	566	566	PO_4_ asymmetric bending ν_4_
472	474	473	-	PO_4_ symmetric bending ν_2_

sh—shoulder.

## Data Availability

Data available on request from the authors.
